# Humus soil as a critical driver of flora conversion on karst rock outcrops

**DOI:** 10.1038/s41598-017-13060-5

**Published:** 2017-10-03

**Authors:** Xiai Zhu, Youxin Shen, Beibei He, Zhimeng Zhao

**Affiliations:** 1 Key Laboratory of Tropical Forest Ecology, Xishuangbanna Tropical Botanical Garden, Chinese Academy of Sciences, Kunming, 650223 China; 2 University of Chinese Academy of Sciences, Beijing, 100049 China

## Abstract

Rock outcrop is an important habitat supporting plant communities in karst landscape. However, information on the restoration of higher biotic populations on outcrops is limited. Here, we investigated the diversity, biomass changes of higher vascular plants (VP) and humus soil (HS) on karst outcrops during a restoration process. We surveyed VP on rock outcrops and measured HS reserved by various rock microhabitats in a rock desertification ecosystem (RDE), an anthropogenic forest ecosystem (AFE), and a secondary forest ecosystem (SFE) in Shilin County, southwest China. HS metrics (e.g. quantity and nutrients content) and VP metrics (e.g. richness, diversity and biomass) were higher at AFE than at RDE, but lower than at SFE, suggesting that the restoration of soil subsystem vegetation increased HS properties and favored the succession of VP on rock outcrops. There was significantly positive correlation between VP metrics and HS amount, indicating that the succession of VP was strongly affected by availability and heterogeneity of HS in various rock microhabitats. Thus, floral succession of rock subsystem was slow owing to the limited resources on outcrops, although the vegetation was restored in soil subsystem.

## Introduction

**Figure 1:**
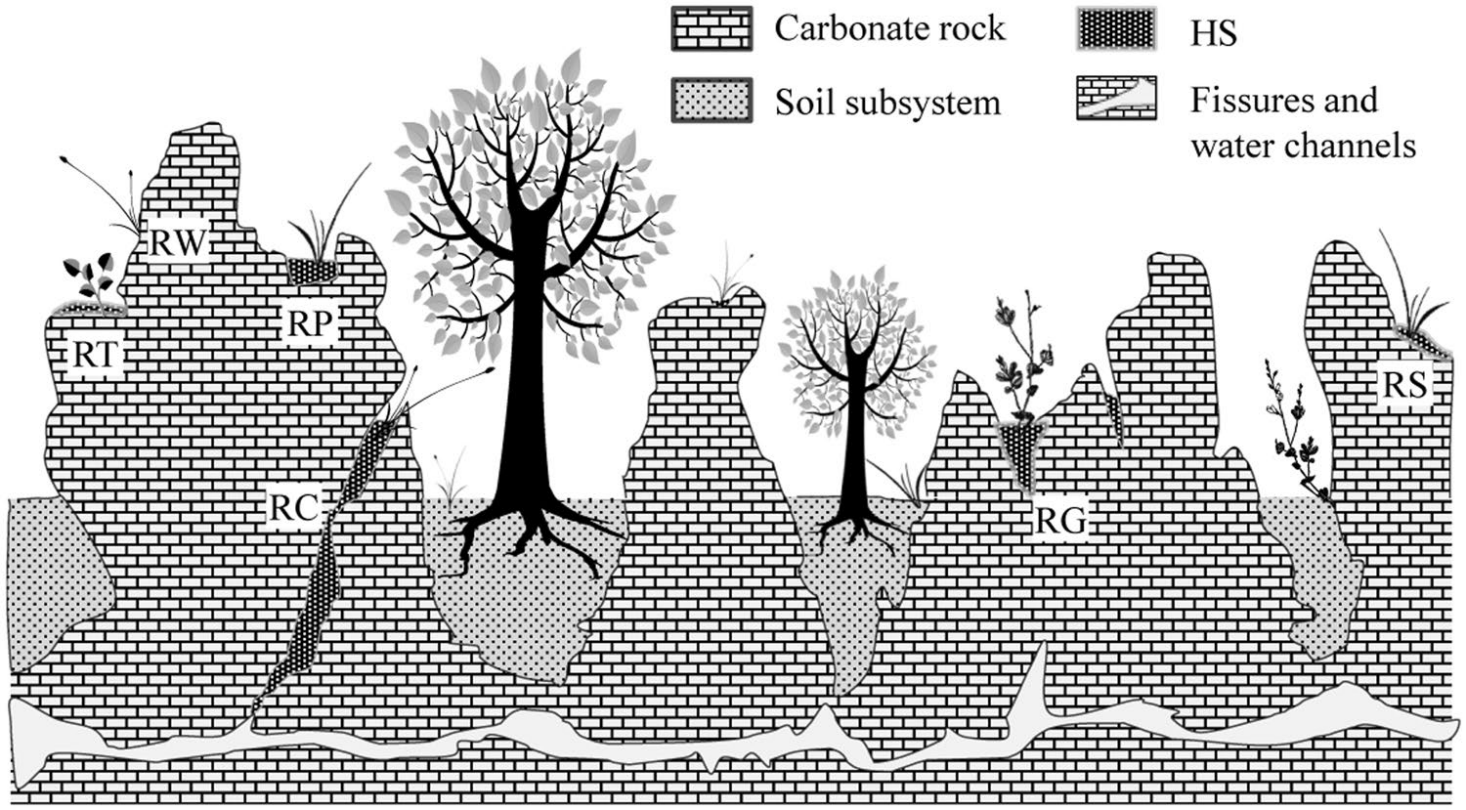
Illustration of the distribution of vascular plants and humus soil on rock outcrops in karst landscapes. RC: rocky crevice, RG: rocky gully, RP: rocky pit, RS: rocky surface, RT: rocky terrace, RW: rocky wall, HS: humus soil on rock outcrops.

Karst landscapes constitute approximately 12–15% of the global terrestrial surface^1^. Depend on the local rainfall and the purity of bedrock, the land surface may consist of various morphologies of rock outcrops and rock-soil patterns^2–4^ (Fig. 1). Human activities (e.g. firewood harvesting and grazing) cause trees growing on soil subsystems to be destroyed leaving rock outcrops exposed. This process is called rocky desertification when the exposed rocks ratio reaches ≥ 30% of the land surface in China^1^, and it is considered a serious environmental problem globally^5^. For example, a recent report revealed that the rocky desertification area is about 1.2 × 10^7^ ha and constitutes 25.6% of total karst terrain in China^1^. Large efforts have been made on researching vegetation degradation/recovery mechanisms of soil subsystems^5,6^, but not on rock outcrops despite their ecological importance.

Carbonate outcrops are formed from different corrosion rates of carbonate rock due to varied chemical compositions, and climatic and geological conditions at different localities^2,4^. Thereby, outcrops gradually form diverse microhabitats (e.g., rocky pit, rocky crevice, rocky surface, rocky gully, etc.) comprising high heterogeneity^7,8^. These microhabitats can intercept and gather soil particles, animal residues, vegetation litter and other exotic substances transported by rainfall and air flow from great distances^9,10^. We refer to these mixed substances as the humus soil on rock outcrops (HS) in this study (Fig. 1).

Plants and their associated soil communities are interlinked and influence each other in ecosystem development^11,12^. The interrelationship between them has been well studied^13^, and remains important for explaining vegetation dynamics^14^. Plant-soil feedbacks can contribute to the coexistence of plant species^15^, alter plant community structure, and act as drivers of vegetation succession^12,16^. Plants can influence soil properties through changing inputs of chemical compounds and organic matter, shaping the hydrological process, changing surface soil temperatures, and providing habitats and resources for rupicolous organisms^17^. Changes to soil chemicals and physical attributes that are caused by plants in turn influence the growth, productivity and reproductive success of individual plants, and the assemblage and floristic composition of these plant communities^14,18^. Flora and scarce soil are common in various rock microhabitats on karst outcrops^9,19^. However, few studies focused on the relationship between the flora and soil in those microhabitats.

Plants on rock outcrops have been proven to substantially contribute to regional biodiversity^20,21^. On rock outcrops, the HS constitutes a suitable growth medium for plants by supplying nutrients, energy, and water sources. Moreover, living organisms (or rock-dwellers) on rock outcrops also fix atmospheric carbon (C) and nitrogen (N) to maintain HS fertility and rock outcrop subsystem productivity^1,22^. Yet there has been, and remains, some confusion concerning the relationship between such microhabitats’ conditions and the rock-dwellers. Particularly, little quantitative research addresses the HS and its ecological function on rock outcrops. In addition, the physical environment of rock subsystems is stressful for plant growth because of low water and nutrients availability, intense solar radiation, great temperature fluctuations, and the shallow soil layer^19,23^. Especially during the dry season in semihumid region (e.g. Shilin), plant growth suffers from severe drought stress^24^. Floral succession in rock subsystems can be considered as an important aspect when evaluating karst degradation and restoration.

The succession of life in (and on) the rock outcrops plays an important role in natural rocky desertification restoration^25,26^. At an early stage of vegetation succession, various types of algae^27–29^, fungi^30^, and cyanobacteria and heterotrophic bacteria colonize where conditions are most favorable^20^. Successively mosses, lichens^21,31,32^, and other higher plants become established in different successional periods^33^. Biological changes of soil subsystems also greatly affect the successional process of rock vegetation. Angelini and Silliman^34^ demonstrated that primary foundation species facilitate secondary foundation species by increasing habitat complexity and quality to enhance biodiversity. The restoration of degraded karst areas may take so long that human interventions, including reforestation, water and soil conservation are required to accelerate the restoration process^5,26^. Previous studies have concentrated on the ecological significance of vegetation restoration in soil subsystems but data concerning floral changes in rock subsystem are limited, and no study addresses the effects of soil subsystem vegetation on plants of rock outcrops.

In this study, we described the vascular plants (VP) and HS on rock outcrops across three karst landscapes, assuming that they were the three typical stages in restoration of rocky desertification. We aim to answer the following questions: (1) How much is the amount of HS on rock outcrops and how does it impact on VP? (2) Do the diversity, density, lifeforms, species composition, and distribution of VP on rock outcrops differ in rocky desertification restoration? (3) Did the rock outcrop subsystem restore following revegetation in soil subsystem? (4) What factors drive rock vegetation succession?

## Results

### HS on rock outcrops

**Figure 2:**
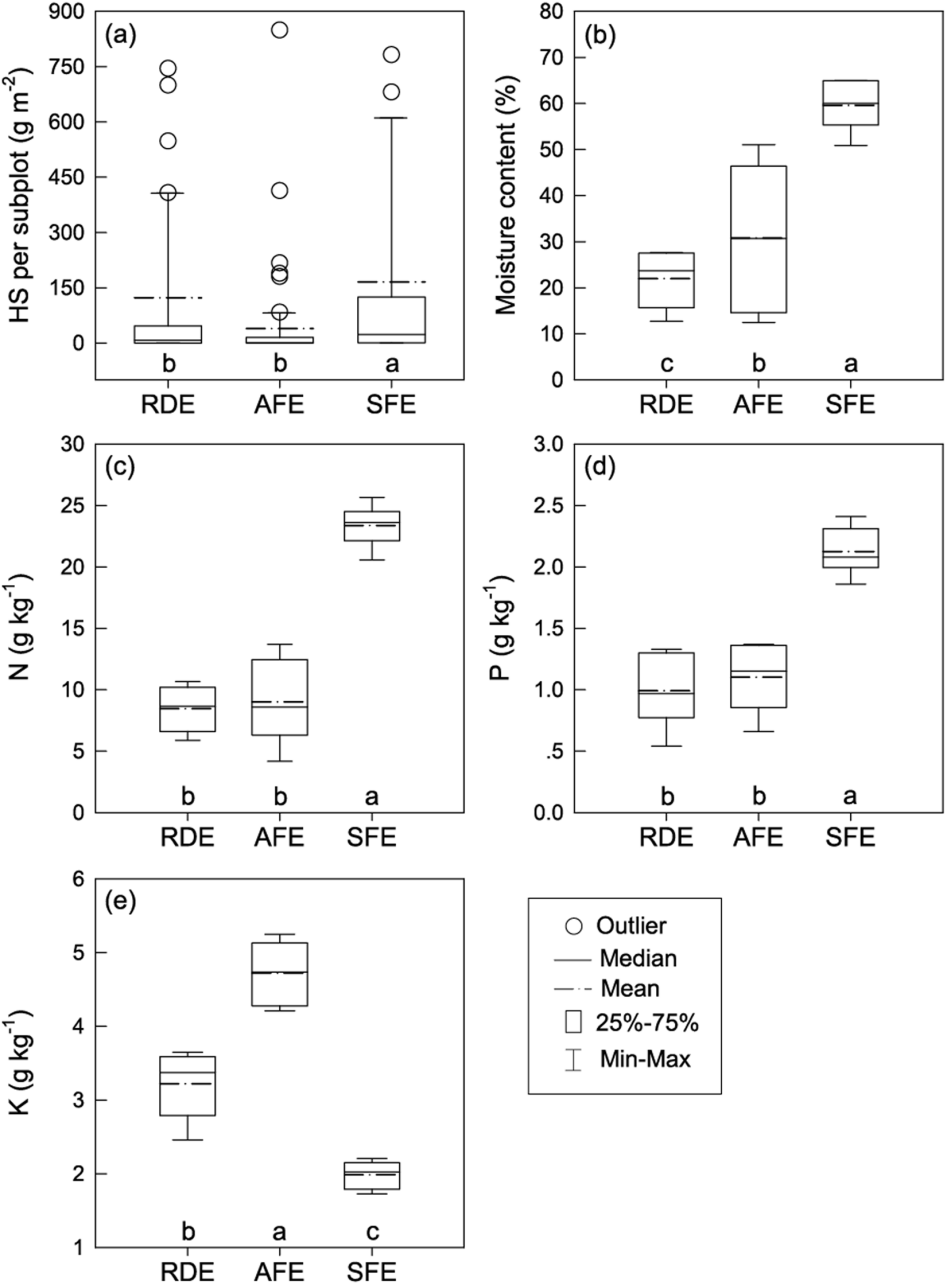
Amount of humus soil and its nutrients content for three study sites in Shilin County, southwest China. Different letters above the x-axis represent significant differences (*P* < 0.05) among different sites. RDE: rocky desertification ecosystem, AFE: anthropogenic forest ecosystem, SFE: secondary forest ecosystem.

The average amounts of HS per unit area was about 10.3 times higher in the SFE (405.54 ± 174.26 g/m^2^) than in the AFE (39.36 ± 16.27 g/m^2^), while for the RDE it was only 3.1 times higher than in the AFE (Fig. 2). On average, the moisture content of the HS showed the following pattern: SFE > AFE > RDE, and it was significantly different among ecosystems. N and P concentration were slight higher at AFE than at RDE, but significant lower than SFE. K concentration was highest at AFE, and lowest at SFE.

### VP on rock outcrops

Sixty-one species, from 58 genera of 37 families were identified in the 180 subplots (Appendix), of which 88.5% were spermatophytes, and 11.5% were ferns. SFE had the highest species richness and RDE had the lowest (Table 1). Seven species (11.4%) occurred in all three ecosystems, while 41 (67.2%) were only identified in one or two ecosystems. Thirteen species were found at both RDE and AFE. Of species unique to an ecosystem, 27 were identified only at SFE, while it was 9 and 5 at AFE and RDE respectively.

**Table 1:** Diversity of VP in three karst outcrops in Shilin County, southwest China.

	Karst ecosystem types			χ^2^	*P*
	RDE	AFE	SFE		
α-diversity	1.38 ± 0.23b	0.92 ± 0.20b	3.53 ± 0.36a	34.53	< 0.001
γ-diversity	21	25	41		
Shannon-Wiener index	0.65 ± 0.09b	0.87 ± 0.10a	1.07 ± 0.06a	15.44	< 0.001
Simpson index	0.37 ± 0.05b	0.50 ± 0.05a	0.56 ± 0.03a	10.15	< 0.01

Values marked with different letters represent significant differences (*P* < 0.05, mean ± S.E.). RDE: rocky desertification ecosystem. AFE: anthropogenic forest ecosystem, SFE: secondary forest ecosystem.

On average, the number of individual per subplot was 34.5 times higher at SFE than that at AFE, and 12.1 times higher than that at RDE (Appendix). Most species (approximately 77.5~96.0%) had less than 50 individuals. *Paraboea neurophylla* and *Selaginella tamariscina* were the dominant species for RDE, *Bidens pilosa* for AFE, and *Pilea pumila var*. *hamaoi* and *Pyrrosia lingua* for SFE.

Average species per subplot ranged from 0.92 to 3.53 and decreased in the order: SFE > RDE > AFE (Table 1), and showed the same trend as γ-diversity. Similarly, the Shannon-Wiener index and Simpson index were both significantly lower for RDE than the other ecosystems. No significant difference was found between AFE and SFE.

**Figure 3:**
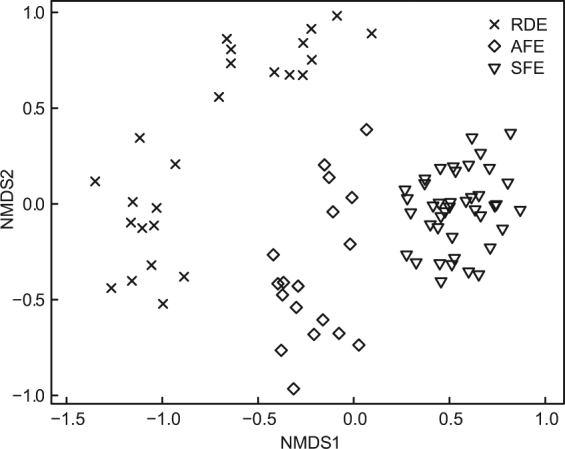
Similarity of VP assemblages in 85 subplots of three karst outcrops in Shilin County, southwest China. Two-dimensional scatterplot of NMDS based on Bray-Curtis index (stress = 0.15; r² = 0.97 for non-metric fit and r² = 0.86 for linear fit of ordination distances with observed dissimilarities). RDE: rocky desertification ecosystem, AFE: anthropogenic forest ecosystem, SFE: secondary forest ecosystem.

The VP communities showed significant differences among different ecosystems in the NMDS ordination scatterplot (Fig. 3). Each of the ecosystems showed distinct VP assemblages. Plots in the SFE were more bunched, while plots in RDE were more scattered.

**Figure 4:**
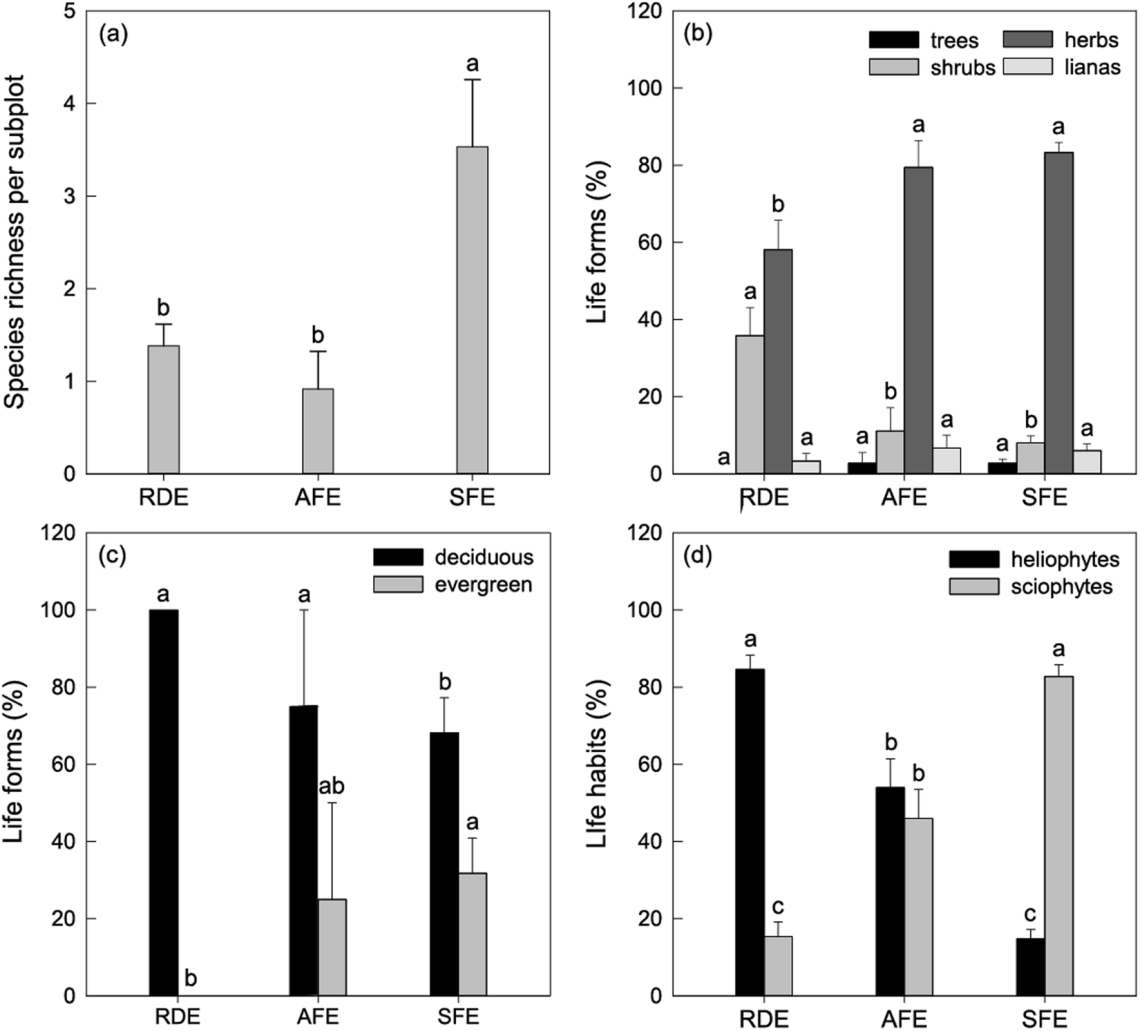
Species richness (**a**) and species composition by life forms (**b**) and (**c**), and life habits (**d**) of VP in subplots for three study sites in Shilin County, southwest China (mean ± S.E. (error bars)). Different letters above the bars represent significant differences (*P* < 0.05) among different sites. RDE: rocky desertification ecosystem, AFE: anthropogenic forest ecosystem, SFE: secondary forest ecosystem.

Herbs were the dominant species in each ecosystem, followed by shrubs, lianas and trees (Fig. 4). More than half (58.1%) of VP were herbs at RDE, and the proportion was significantly lower than at the other two ecosystems. Yet, the proportion of shrubs at RDE was statistically significantly higher than at AFE and SFE. The percentages of lianas were surprisingly low for the three ecosystems. A few trees occurred at AFE and SFE but not at RDE. Generally, deciduous species were richer than evergreen. The proportional representation of deciduous and heliophyte species among ecosystems showed a decreasing order: RDE > AFE > SFE. SFE had the greatest evergreen and sciophytes, followed by AFE, and RDE had the lowest. In addition, the heliophytes were proportionally 5.6 times greater than sciophytes at RDE, while the opposite was true at SFE.

### The relationship between plant and physical factors

Correlation analysis showed that the numbers of individuals, species richness, and biomass of VP were positively related to the amount of HS, canopy coverage, rock outcrops ration, and relative humidity (Table 2). HS on rock outcrops was the most important variable to explain the number of individuals, species richness, and biomass.

**Table 2:** The Spearman’s correlation coefficients (r) and their significance levels between factors and individuals, species richness and biomass of VP. VP: vascular plant, HS: humus soil. *Means P < 0.05, **means P < 0.01.

Factors	VP		
	Individuals	Species richness	Biomass (g/m²)
HS (g/m²)	0.776**	0.749**	0.787**
Canopy coverage (%)	0.274**	0.293**	0.113
Rock outcrops ratio (%)	0.472**	0.448**	0.524**
Air temperature (°C)	−0.398**	−0.396**	−0.255**
Relative humidity (%)	0.205**	0.206**	0.072
Photosynthetically active radiation (mol m^−2^ s^−1^)	−0.272**	−0.253**	0.085

**Figure 5:**
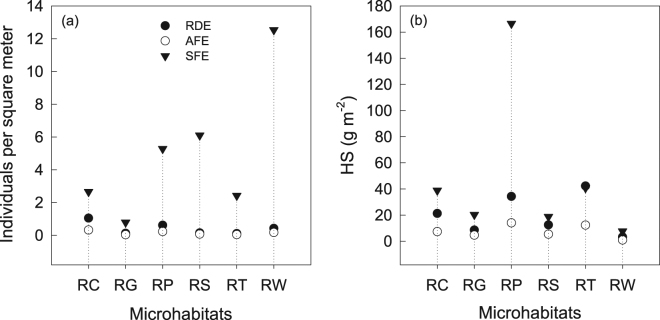
VP individuals (**a**) and HS (**b**) in microhabitats per unit area for three study sites in Shilin County, southwest China. RC: rocky crevice, RG: rocky gully, RP: rocky pit, RS: rocky surface, RT: rocky terrace, RW: rocky wall. RDE: rocky desertification ecosystem, AFE: anthropogenic forest ecosystem, SFE: secondary forest ecosystem.

A large proportion of vascular individuals were biased in favor of the RW, RS and RP microhabitats at SFE (Fig. 5a). Yet most individuals were recorded in RC and RP at RDE and AFE. The VP individuals in any of the microhabitat per square meter at SFE were higher than at AFE and RDE. A vast majority of HS was accumulated in RP at SFE, while HS was mainly stored in RT and RP at RDE. Only a small subset could be found at RW (Fig. 5b). In particular, the HS at RP for SFE was evidently higher than in the other two ecosystems.

## Discussion

Over the large karst area, such practice as reforestation in the soil subsystem was usually adopted to control rocky desertification and accelerate ecosystem restoration^5,35,36^. Much research has focused on soil-vegetation restoration processes^5,6,23^. However, there is no information concerning the rock-vegetation conversion and its relationship with the soil retained on rock outcrops. Furthermore, there is no research addressing the changing pattern of VP and HS on the highly fragile rock subsystem during the restoration of vegetation in soil subsystem. Our data indicated that a divers VP can be found on rock outcrops with declining density at the various microhabitats holding different amounts of HSs in the SFE. With the removal of forest from the soil sub-ecosystem, VP and HS will be highly diminished. On the other hand, the loss of VP and HS will be reversed with the restoration of tree plantations in the soil subsystem.

Vascular flora on rock outcrops have a broad distribution in a range of habitats from savannas to humid tropical forests^19^. However, little research considers the changing pattern along the vegetative gradient on the nearby soil subsystem. Our results suggested that VP metrics obviously changed with the degradation and restoration of soil subsystem vegetation (Fig. 4, Table 1). When the canopy coverage in soil subsystem at SFE was reduced to that of RDE (Table 3), the species richness of VP lost 48.8% and diversity indices dropped by approximately 50% (Table 1). Moreover, NMDS ordination analysis showed a strong separation between forest (e.g. AFE and SFE) and non-forest (e.g. RDE) sites (Fig. 3). The VP were characterized by calcification and xerophilization at RDE, such as dense growth (*Paraboea neurophylla*), desiccation-tolerant (*Selaginella tamariscina*), or deep rooted (*Ficus tikoua*)^37^. Similarly, the VP also changed with the restoration of soil subsystem vegetation. These changes in community structure and composition followed a trend towards SFE but remained different from it. Certain life forms (i.e., shrubs, herbs, deciduous and evergreen plants) and space distribution of VP at AFE were in an intermediate stage of developmental sequence between RDE and SFE.

**Table 3:** Environmental conditions and main meteorological information (mean ± S.E.) of three study sites in Shilin County, southwest China. Air temperature, relative humidity and photosynthetically active radiation were the average of data measured on three sunny days (during the period of 12:00~14:00) in both the rainy season (October 2014) and dry season (January 2015). The different lowercase letters indicate significant differences between the different sites (*P* < 0.05).

	Rocky desertification ecosystem (RDE)	Anthropogenic forest ecosystem (AFE)	Secondary forest ecosystem (SFE)
Coordinates	24°51′25.92″N 103°19′49.44″E	24°49′49.8″N 103°19′32.52″E	24°38′52.08″N 103°20′18.69″E
Slope directions	WE	NE	NE
Slope (°)	10°~30°	15°~20°	30°~45°
Altitude (m)	1789	1927	1776
Stand age (year)	< 20	20	> 50
Height of soil subsystem vegetation (m)	< 1.5	1~5	2~15
Rock outcrops ratio (%)	44.71 ± 4.29a	36.35 ± 3.39a	32.41 ± 4.10a
Canopy coverage (%)	5.76 ± 0.30c	35.03 ± 2.41b	65.43 ± 2.78a
Air temperature (°C)	21.36 ± 0.15a	20.44 ± 0.29b	17.31 ± 0.14c
Relative humidity (%)	58.97 ± 1.00c	64.74 ± 1.55b	73.47 ± 0.88a
Photosynthetically active radiation (mol m^−2^ s^−1^)	70.05 ± 2.73a	51.91 ± 1.93b	11.71 ± 1.20c

HS also changed following the soil subsystem vegetation degradation, as did the restoration process, in both quantity and quality. Average amount and nutrient content of HS at RDE were significantly lower than at SFE (Fig. 2). Almost 30% quantity, 35% moisture, 33% N, and 40% P in HS of SFE accounted for that at RDE. Nevertheless, the moisture and nutrients content (except K) at AFE were slightly higher than those of RDE. In addition, the HS amount and its nutrient concentration at AFE sharply decreased compared with SFE, like the variation pattern of VP individuals per square meter and microhabitats. These results indicated that the VP and HS were highly sensitive to the vegetation changes on nearby soil subsystems, and neither of them have improved substantially despite the successful vegetation restoration in soil subsystems.

The availability and heterogeneity of HS are important factors for the slow succession of VP since a strong correlation was found between HS and VP metrics (Table 2). Our results showed that the individuals and species richness of VP were significantly positive correlated with the amount of HS on rock outcrops. Moreover, the variation of species richness was similar to that of HS among three sites (Figs 2, 4). Thus HS, to some extent, may induce the successful establishment of VP by supplying sufficient water and preventing nutrients leaching into runoff water or seeping through the fissures, and also acts as a medium for roots proliferation. The amount and quality differences in HS patches would increase the heterogeneity of substrate fertility, and significantly influence karst biodiversity. However, because of the limited retention capacity of HS on rock outcrops^10^, much water, redundant organic carbon and nutrients were transported to nearby soil subsystems in local karst landscape^1,38^.

The low HS quantity can be attributed to the extremely slow rate of soil formation because little residue results from carbonate rock weathering^39^. On average, it takes 300–73800 years to form 1 cm thick soil based on different estimation methods involving the dissolution test of carbonate rock^39^. The magnitude of quantity and quality in HS, and associated ecological function at AFE cannot reach the levels within the regional natural forests (e.g. SFE). Therefore, the floral succession on rock outcrops remained slow in plantation forest. Additionally, HS tended to amass at the “funnel” shaped microhabitats (RC and RP), whereas few residua were detained at the rain-washed places (Fig. 5b). The finite and shallow substrate layer, combined with low availability of resources made it difficult for flora to develop in rock subsystem. Perhaps this could help explain the species richness of our carbonate outcrops (61) being lower than other outcrops, such as 86 for granite outcrops^40^, and 142 for crystalline outcrops^19^. Research showed that soil communities changed over time to favor late successional plant species^41^, although the soil depth had litter influence on community structure during the initial years of restoration^42^. Plant-soil feedbacks, the successful drivers of succession^12^, play a vital role in explaining slow outcrops flora succession. In return, our study provided an obvious evidence that availability and heterogeneity of resources (e.g. nutrients and soil heterogeneity) have a strong influence on plant community structure during the restoration of rock desertification.

In addition, the flora on rock outcrops exhibits interesting patterns of distribution and affinity to the substratum^40^. Porembski^43^ reported many species on inselbergs distributed in ephemeral flush plot and a shallow depression with more nutrients and soil. Our study showed that distinctive species were usually found in diverse microhabitats with a shallow soil layer, forming a patchy size distribution ranging from a few centimeters across to hundreds of square centimeters. This agrees with other studies where numerous individual plants were biased in favor of rocky habitats where considerable amounts of HS was easily accumulated^10,44^. Outcrops which appear bare at a distance, however, can bear many vascular plant species, some of which have the striking ability to grow on a surface with almost no soil^40^. SFE reflected this; there was very little HS in RW but many VP. More than 160 g/m² of HS occurred at RP with only relatively few individuals (Fig. 5). This uncommon phenomenon could suggest that the species diversity in rock vegetation may be influenced by other factors (e.g., microclimate, or physiological characteristics of plants).

Many factors are likely to influence the succession of plants in rocky habitats. Angelini and Silliman^34^ observed that vegetation species initially settled in the community (e.g., soil subsystem vegetation) which may facilitate the establishment of subsequent species by improving the habitat. Clements *et al*.^45^ claimed the presence of karst microhabitats supported high floral diversity. Both primary communities and diverse topographies result in a difference of microclimate, and further affect the structure and distribution of VP. Additionally, seed and establishment limitations restrict plant recruitment at early stages during ecological restoration^36,46^. Compared with soil subsystem vegetation, more stressful establishment conditions (e.g., low coverage, scanty soil, and intensive human intervention) resulted in slow flora succession on outcrops.

Ecological restoration is not just a matter of planting trees, it involves assisting the restoration of a damaged and destroyed ecosystem^47^. Rock outcrop is a critical component of karst landscapes and typically supports the vast majority of its entire floristic diversity^45^, even in the severe environment of RDE. Certain attempts have been made to alleviate the rate of environmental degradation in the vulnerable karst area^6,19^, and have made good progress^5^. Most of the eco-efficiency indexes that evaluate rocky desertification control, mainly come from soil subsystem vegetation, such as coverage, biomass, and diversity indexes^48^. Due to the different VP variation pattern during rocky desertification restoration, its succession should be taken into consideration when constructing an evaluation index system of rocky desertification restoration, especially in areas with a high rock-to-soil area ratio.

## Methods

### Study area

This study was performed in Shilin County (24°30′–25°03′N, 103°10′–103°40′E), Yunnan Province, southwest China, where karst landforms are composed of Permian, Carboniferous and Devonian carbonate rocks^49^. The karst landscapes consist of rock gaps, rock ditches, small rock caves and rock slots. Altitudes range from 1600 to 2200 m above sea level. A subtropical plateau monsoon climate prevails with a mean annual precipitation of 967.9 mm, 80–88% of which falls between May and October. Mean annual temperature is 16.2 °C, mean maximum temperature of the warmest month (July) is 20.7 °C, and mean minimum temperature of the coldest month (January) is 8.2 °C ^36^. Red earth and calcareous soil are the primary soil types.

Three typical local ecosystems were selected for this study:

1. A rocky desertification ecosystem (RDE), where most of the trees of the primary forest were removed or cut because of human activities (e.g., firewood harvesting, animal grazing), followed by the establishment of shrubs and herbs, such as *Sophora viciifolia*, *Spiraea salicifolia*, *Diospyros duetorum*, *Bidens pilosa*, *Heteropogon contortus*, *Themeda triandra*. Rock outcrops are exposed. The rock surface is covered by cyanobacteria film, and a few VP occur in microhabitats formed on rock outcrops.
2. An anthropogenic forest ecosystem (AFE), with trees including *Pinus yunnensis*, *Koelreuteria paniculata*, *Photinia* × *fraseri*, and *Pyracantha fortuneana* which were planted after a land preparation on a RDE site in 2005. Currently, these trees are higher than most of rock outcrops. The site is 4 km away from the RDE. Tian *et al*.^29^ investigated the species and communities of epilithic cyanobacteria films in this area. There are more VP on rock outcrops compared with RDE. No restoration measures were applied to the vegetation on rock subsystem and they are the result of succession.
3. A secondary forest ecosystem (SFE), is an evergreen broadleaved forest dominated by *Neolitsea homilantha*, *Olea yunnanensis*, *Cyclobalanopsis glaucoides* and *Pistacia weinmannifolia*, with mixed deciduous species such as *Albizia mollis*, *Carpinus mobeigiana* and *Pistacia chinensis*. It was a god forest of a local village, the forest is dense, has a clear vertical stratification, and outcrops are covered by the tree canopy. Cyanobacteria, lichen, bryophytes, and many VP live on the rocks^50^. The site is 26.5 km away from RDE, within the natural reserve of Shilin Geopark.

The specific degradation process of RDE is difficult to ascertain for the complicated reasons^51^. For AFE, the soil subsystem vegetation has almost been restored after a period of more than 20 years. The time was confirmed by the record of Stone Forest Scenic Area Administration. There were no trees in soil subsystem before restoration according to the description of local people, although there was no detailed record about it. The previous situation of AFE could be regarded as same as RDE. Vegetation is well preserved in the SFE with slight human disturbance. All three ecosystems are located on the same karst rock base, and have similar geographical features. We conceptualized the RDE as the degradation stages of SFE, and the AFE and SFE as the ecological restoration stages of RDE by employing a space-for-time substitution method^52^. The coordinates and environmental conditions of the study sites are shown in Table 3.

### Plot design

**Figure 6:**
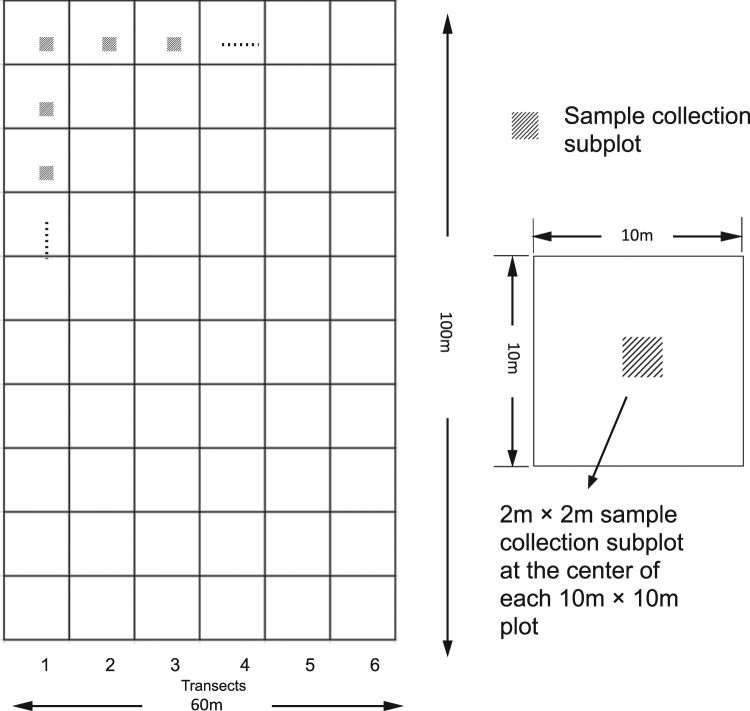
Illustration of sampling designs in study sites.

Comprehensive fieldwork was undertaken between October and November 2014. Six transects of 10 m wide were established along the hill slope within each of the three ecosystems and then each transect was divided into 10 plots with plastic line (Fig. 6). At the center of each plot (10 m × 10 m), a 2 m × 2 m sample collection subplot was established. There were 180 subplots in total for the three ecosystems.

### Vascular plant identification and classification

Within each 2 m × 2 m subplot, all vascular species (VP) growing on rock outcrops were identified. Some plants were too small to identify. We only recorded the recognizable individuals more than one centimeter high. Next, all the recorded VP were collected, sealed within bags and brought back to laboratory, dried for 72 h at 70 °C to attain constant weight to calculate biomass. Such biomass obtained for the rock outcrops must be regarded as conservative. This is because a few shrubs and lianas had roots extending into soil, but only the part that was growing on rock outcrops was collected and counted as biomass.

Based on our investigation and analysis, the VP were grouped into life forms of trees, shrubs, herbs and lianas^53^, and further grouped into deciduous and evergreen, and into heliophyte and sciophyte^54^ for comparison among the three ecosystems.

### Humus soil collection and chemical determination

The microhabitats on rock outcrops were divided into six categories: rocky crevice (RC), rocky gully (RG), rocky pit (RP), rocky surface (RS), rocky terrace (RT), rocky wall (RW)^8^. Their specific features are shown in Table 4. The numbers of microhabitat type within each subplot were recorded. A maximum of three for each microhabitat type were selected to collect HS depending on the total individuals counted. As much HS as possible was collected, stones and other impurities were removed, and it was then air-dried and weighed. The total amount of HS for each microhabitat in each subplot was multiplied by the number of each microhabitat type to calculate the mean weight of replicates.

**Table 4:** General features and types of microhabitat on rock outcrops.

Microhabitat	Rock body	Vegetation	Humus soil	Soil humus water
Rocky crevice	10–300 cm high, 1–20 cm depth, 0.002–0.2 m^2^	Many ferns and shrubs, few trees in some crevices	Weathering and humification into considerable soil humus, thickness of 3–8 cm, rock deep groove shape distribution	Rainfall, runoff and litter layer infiltration water, humid throughout the year
Rocky gully	0–200 cm high, 5–30 cm depth, 0.02–0.16 m^2^	Limited herbs and ferns	Weathering and humification formed less soil humus, thickness of 2–5 cm, semi-decomposed layer, shallow groove shape	Rainfall, runoff and litter layer infiltration water, more humid
Rock pit	1–20 cm depth, 0.0025–0.09 m^2^	Many herbs, plant height 2–10 cm	Large amount of soil humus formation mainly by humification, 1–10 cm thickness, cylinder and funnel shape	Rainfall, runoff and litter layer infiltration water, mainly water collection, moisture saturation in rainy season
Rocky surface	20–300 cm high, 10–60°, 0.04–2 m^2^	A large amount of mosses and lichens, few herbs	Litter decomposition and moss residues constituted little soil humus, thickness of 0–2 cm, clustered distribution	Rainfall and water held by mosses layer, desiccation in dry season, humid in rainy season
Rocky terrace	50–100 cm high, 0–10°, 0.04–0.5 m^2^	Many herbs and lianas, few ferns, plants’ height 1–15 cm	Greater soil humus development via rock weathering and humification, 0–5 cm thickness, patchy distribution	Rainfall, runoff and litter layer infiltration water, relatively humid all year
Rocky wall	20–300 cm high, 70–90°, 0.01–1 m^2^	Considerable mosses and herbs, few lichens and ferns	Rock weathering formed limited soil humus, thickness less than 1 cm, point distribution	Rainfall and water held by mosses layer, humid in rainy season

The HS samples from each transect were combined into one sample (six samples for each ecosystem), passed through a 0.25 mm sieve, and subjected to chemical analysis. Total N was determined using a Vario MAX CN elemental analyzer. The P and K content were determined with an ICP-AES (iCAP6300, Thermo Fisher Scientific U.S.A) after mixing with HNO_3_-HClO_4_.

### Statistical analyses

VP diversity was estimated using (1) α-diversity, evaluated as species richness per subplot, (2) γ-diversity, the total species numbers in each karst outcrops, (3) Shannon-Wiener index, (4) Simpson index^55^.

We calculated VP similarities using Sorensen’s similarity index^55^. Moreover, to estimate the differences in VP community across ecosystem types, a similarity matrix of 85 plots (eliminated without plants) × 61 species was subjected to non-metric multidimensional scaling (NMDS) with function *metaMDS* ^56^ in the package *vegan* ^57^ in R 3.3.2 ^58^. NMDS is a good ordination method because it can use rank information and map ranks non-linearly onto ordination space to measure community dissimilarities based on species data.

One-way ANOVA, followed by a Tukey test for multiple pair-wise comparison, were used to assess the differences in diversity of VP among the three study sites. All data were checked for homogeneity of variances using Bartlett’s test and normality using the Shapiro-Wilk test and when the assumptions could not be satisfied after transformation, comparisons of non-parametric data were made using the Kruskal-Wallis test and Wilcoxon rank sum test. We performed all calculations and statistical analyses in R 3.3.2 ^58^.

Kruskal-Wallis test and Wilcoxon rank sum test were used to assess the difference in the quantity and nutrients concentration of HS among ecosystems. Spearman’s correlation coefficients were used to express the relationship between physical factors and number of individuals, species richness and biomass.

## Electronic supplementary material

Supplementary information
