# Supplementary material for: Humus soil as a critical driver of flora conversion on karst rock outcrops
Source: Sci Rep. 2017 Oct 3;7:12611. doi: 10.1038/s41598-017-13060-5 (PMC5626776; doi:10.1038/s41598-017-13060-5)
Supplement: Supplementary file 1 — Supplementary information [file 41598_2017_13060_MOESM1_ESM.pdf]

Humus soil as a critical driver of flora conversion on karst rock outcrops

Xiai Zhu[1,2], Youxin Shen[1*], Beibei He[1,2], Zhimeng Zhao[1,2]

[1] Key Laboratory of Tropical Forest Ecology, Xishuangbanna Tropical Botanical Garden, Chinese Academy of Sciences, Kunming 650223, China

[2] University of Chinese Academy of Sciences, Beijing 100049, China

*Correspondence and requests for materials should be addressed to Y.S. (email: yxshen@xtbg.ac.cn)

Appendix The microhabitats, frequencies, total and average number of individuals in the three different karst outcrops in the Shilin County, southwest China (mean ± S.E.). RC: rocky crevice, RG: rocky gully, RP: rocky pit, RS: rocky surface, RT: rocky terrace, RW: rocky wall. RDE: rock desertification ecosystem. AFE: anthropogenic forest ecosystem, SFE: secondary forest ecosystem.

Species	Number of individuals (N)	Average number of individuals per subplot (N /60)	Frequencies（%）	Microhabitats
RDE				
Paraboea neurophylla	171	2.85 ± 0.96	20.00	RC, RG, RP, RT
Selaginella tamariscina	166	2.77 ± 1.23	15.00	RC, RP, RT, RW
Spiraea martinii	77	1.28 ± 0.45	21.67	RC, RP, RS
Tripogon bromoides	62	1.03 ± 0.45	15.00	RC, RW, RT
Asplenium varians	44	0.73 ± 0.40	15.00	RC, RG, RP
Aleuritopteris duclouxii	13	0.22 ± 0.14	6.67	RC
Heteropogon contortus	11	0.18 ± 0.08	5.00	RC, RS
Rhamnus leptophylla	11	0.18 ± 0.13	10.00	RC
Eulaliopsis binata	6	0.10 ± 0.10	1.67	RP
Digitaria sanguinalis	6	0.10 ± 0.10	1.67	RP
Rubus mesogaeus	3	0.05 ± 0.04	3.33	RC
Pteridium aquilinum var. latiusculum	3	0.05 ± 0.05	3.33	RC
Arthraxon hispidus	3	0.05 ± 0.05	3.33	RP
Bidens pilosa	3	0.05 ± 0.04	1.67	RP, RS
Pilea pumila var. hamaoi	3	0.05 ± 0.05	1.67	RS
Rubia oncotricha	3	0.05 ± 0.04	1.67	RC, RP
Eupatorium adenophora	2	0.03 ± 0.02	3.33	RC
Themeda japonica	2	0.03 ± 0.02	3.33	RW
Clematis florida	1	0.02 ± 0.02	1.67	RW
Ficus tikoua	1	0.02 ± 0.02	1.67	RC
Cynoglossum amabile	1	0.02 ± 0.02	1.67	RS
Total	592	9.87 ± 2.57		
AFE				
Bidens pilosa	89	1.48 ± 0.73	13.33	RC, RG, RP, RW

Tripogon bromoides	26	0.43 ± 0.26	6.67	RC, RT, RW
Heteropogon contortus	22	0.37 ± 0.18	11.67	RC, RP, RS
Asplenium varians	21	0.35 ± 0.24	8.33	RC, RW
Hypodematium crenatum	7	0.12 ± 0.08	3.33	RW,
Rubia oncotricha	6	0.10 ± 0.05	6.67	RC, RG
Pyrrosia lingua	4	0.07 ± 0.07	5.00	RC
Achyranthes bidentata	4	0.07 ± 0.04	3.33	RC, RS
Digitaria sanguinalis	4	0.07 ± 0.05	1.67	RC, RT
Tagetes patula	3	0.05 ± 0.05	1.67	RG
Pteridium aquilinum var. latiusculum	3	0.05 ± 0.05	1.67	RP
Eupatorium adenophora	2	0.03 ± 0.02	3.33	RS
Arthraxon hispidus	2	0.03 ± 0.03	3.33	RC
Cynoglossum amabile	2	0.03 ± 0.02	3.33	RW
Imperata cylindrica	2	0.03 ± 0.02	1.67	RC
Capillipedium assimile	1	0.02 ± 0.02	1.67	RC
Rubus mesogaeus	1	0.02 ± 0.02	1.67	RP
Eulaliopsis binata	1	0.02 ± 0.02	1.67	PC
Albizia mollis	1	0.02 ± 0.02	1.67	RP
Aleuritopteris duclouxii	1	0.02 ± 0.02	1.67	RC
Sonchus oleraceus	1	0.02 ± 0.02	1.67	RC
Micromeria biflora	1	0.02 ± 0.02	1.67	RS
Lespedeza juncea	1	0.02 ± 0.02	1.67	RC
Gerbera delavayi	1	0.02 ± 0.02	1.67	RT
Sida szechuensis	1	0.02 ± 0.02	1.67	RC
Total	207	3.45 ± 1.05		
SFE				
Pilea pumila var. hamaoi	2686	44.77 ± 15.37	45.00	RC, RS, RP, RT, RW
Pyrrosia lingua	2159	35.98 ± 13.91	60.00	RC, RP, RW
Peperomia tetraphylla	883	14.72 ± 6.88	30.00	RS, RW
Asplenium varians	367	6.12 ± 1.30	55.00	RC, RS, RW
Paraboea neurophylla	232	3.87 ± 2.88	13.33	RC, RS, RW
Lepisorus thunbergianus	171	2.85 ± 0.75	35.00	RC, RP, RW
Corydalis edulis	166	2.77 ± 1.98	3.33	RP, RT, RW
Eupatorium adenophora	163	2.71 ± 1.27	13.33	RC, RS, RW
Aleuritopteris duclouxii	76	1.27 ± 0.73	1.67	RC, RT
Pouzolzia sanguinea	73	1.22 ± 0.40	13.33	RP, RG, RS
Lonicera japonica	35	0.58 ± 0.36	5.00	RG, RS, RW
Cyclobalanopsis glaucoides	29	0.48 ± 0.33	5.00	RP, RS
Sinocrassula indica	21	0.35 ± 0.23	3.33	RW
Digitaria sanguinalis	14	0.23 ± 0.20	1.67	RS
Millettia bonatiana	12	0.20 ± 0.12	1.67	RP, RW
Bidens pilosa	7	0.12 ± 0.08	1.67	RS
Phymatopteris griffithiana	5	0.08 ± 0.06	1.67	RG
Zanthoxylum scandens	4	0.07 ± 0.04	1.67	RG
Nandina domestica	4	0.07 ± 0.04	5.00	RC, RP
Dioscorea arachidna	4	0.07 ± 0.05	5.00	RS
Neolitsea homilantha	4	0.07 ± 0.07	3.33	RC, RS
Fallopia multiflora	4	0.07 ± 0.05	1.67	RS
Albizia mollis	3	0.05 ± 0.04	1.67	RS
Ophiopogon bodinieri	3	0.05 ± 0.03	3.33	RS
Dioscorea hemsleyi	3	0.05 ± 0.02	5.00	RC
Smilax china	2	0.03 ± 0.03	3.33	RC
Myrsine africana var. acuminata	2	0.03 ± 0.02	1.67	RC, RP

Achyranthes aspera	2	0.03 ± 0.02	1.67	RC, RP
Rubia oncotricha	2	0.03 ± 0.02	3.33	RC
Trachelospermum bodinieri	2	0.03 ± 0.02	3.33	RP, RS
Hedychium spicatum	1	0.02 ± 0.02	1.67	RC
Anredera cordifolia	1	0.02 ± 0.02	1.67	RW
Pistacia weinmannifolia	1	0.02 ± 0.02	1.67	RG
Ainsliaea bonatii	1	0.02 ± 0.02	1.67	RW
Stemona mairei	1	0.02 ± 0.02	1.67	RS
Myrsine semiserrata	1	0.02 ± 0.02	1.67	RC
Senecio scandens	1	0.02 ± 0.02	1.67	RC
Hypodematium crenatum	1	0.02 ± 0.02	1.67	RS
Arthraxon hispidus	1	0.02 ± 0.02	1.67	RC
Clematis florida	1	0.02 ± 0.02	1.67	RC
Sedum sp.	1	0.02 ± 0.02	1.67	RS
Total	7149	119.15 ± 32.73		
